# Pruritus as an early sign of abnormal scarring in the re‐epithelialising phase of Toxic Epidermal Necrolysis

**DOI:** 10.1002/ski2.217

**Published:** 2023-02-15

**Authors:** Aaron J. Frederiks, Jasmin D. Korbl, Nima Mesbah Ardakani, Fiona M. Wood, Bernadette Ricciardo

**Affiliations:** ^1^ Department of Dermatology Fiona Stanley Hospital Murdoch Western Australia Australia; ^2^ Faculty of Health and Medical Sciences School of Medicine The University of Western Australia Perth Western Australia Australia; ^3^ Department of Anatomical Pathology PathWest Laboratory Medicine QEII Medical Centre Perth Western Australia Australia; ^4^ School of Pathology and Laboratory Medicine The University of Western Australia Perth Western Australia Australia; ^5^ College of Science, Health, Engineering and Education Murdoch University Perth Western Australia Australia; ^6^ State Burns Service Fiona Stanley Hospital Murdoch Western Australia Australia; ^7^ Burn Injury Research Unit The University of Western Australia Perth Western Australia Australia

## Abstract

We present a 28‐year‐old remote‐living male who presented to our dermatology clinic with increasing pruritus over his torso and limbs in the context of a recent admission for Toxic Epidermal Necrolysis (TEN) secondary to paliperidone depot. Our case demonstrates that pruritus in the re‐epithelialising phase of TEN may be a sign of abnormal scarring. Early assessment and measurement for compression garments is recommended.

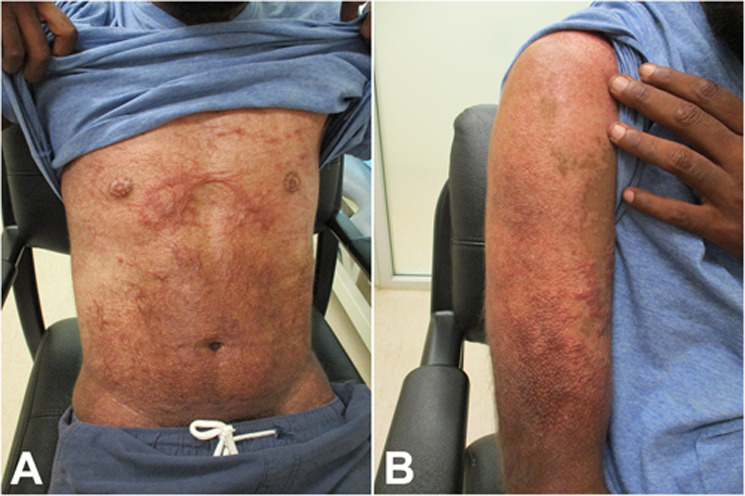

## ETHICS STATEMENT

The study has been approved by the appropriate body and written informed consent has been obtained from the patient the subject of the manuscript.

1

Toxic Epidermal Necrolysis (TEN) presents with sloughing and blistering of mucocutaneous epithelium over more than 30% of the body surface area and is precipitated by a rare, severe drug reaction.^1^ Patients can face acute complications and significant morbidity from delayed ocular, mucocutaneous, psychological and other internal sequelae.^1^

A 28‐year‐old remote‐living male presented to our dermatology clinic with increasing pruritus over his torso and limbs. This was on a background of TEN secondary to paliperidone depot, for which he had been discharged 12 days earlier following a 1‐month admission, including 16 days in the intensive care unit. Maximum epithelial detachment (>50% BSA) was seen on day 6, re‐epithelialisation commenced day 9, pruritus over the anterior torso and proximal limbs was first reported on day 18 and re‐epithelialisation was complete by day 25.

Additional discharge diagnoses included cutaneous dermatophyte infection, irritant contact phyto‐dermatitis, pellagra, folate and vitamin D deficiency, and secondary polymicrobial bacterial infection of the skin. Following discharge, our patient reported no new topical products or systemic medications. His medical history included paranoid schizophrenia and self‐harm.

Examination revealed erythematous re‐epithelialised skin with follicular prominence over the anterior torso and proximal limbs (corresponding to the sites of early pruritus), in a Fitzpatrick skin phototype VI patient. Excoriations and erosions with yellow crusting were noted over the upper torso. Nikolsky's sign was negative. The mucosa were clear.

**FIGURE 1:**
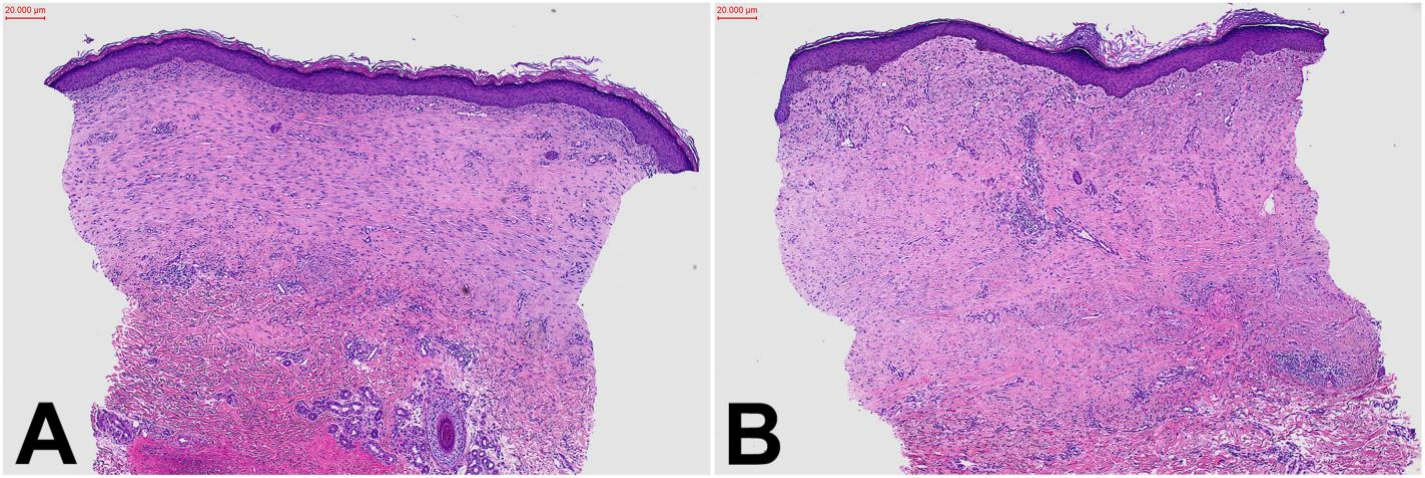
((a) and (b)) Medium power microscopic examination of punch biopsies from 2 different anatomical sites demonstrates areas of superficial to mid‐dermal scarring with overlying flattened regenerative epidermis (H&E, x40)

The clinical impression was of early pruritus and subsequent hypertrophic scarring, with excoriation and secondary bacterial infection. He was admitted for assessment and symptom control with potent corticosteroid ointment wet dressings and antibiotics. Skin punch biopsies from the abdomen and right arm were performed. Microscopic examination revealed superficial to mid dermal scarring characterised by arrays of collagen fibres parallel to the epidermal surface with scattered perpendicularly orientated blood vessels (Figure 1 a,b). The overlying epidermis had a regenerative appearance.

**FIGURE 2:**
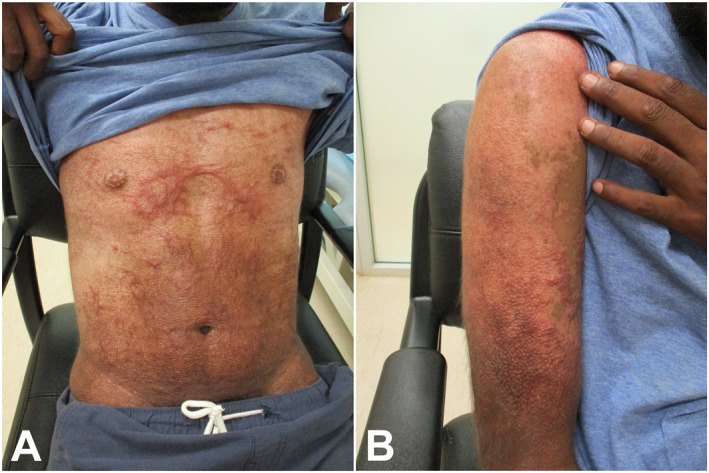
((a) and (b)) Hypertrophic scarring on the torso and right arm of a 28‐year‐old Aboriginal male following Toxic Epidermal Necrolysis secondary to paliperidone

Topical therapies provided limited relief. Cryotherapy could not be performed during his admission given the erosions due to excoriation. The patient was referred to the burns service and measured for compression garments. Silicone sheets were not appropriate for the patient given the humidity and heat of his tropical semi‐arid location together with the challenges of monitoring for any adverse sequelae of the silicone given his remote location. At telehealth follow‐up 2 months after discharge, his arm and torso demonstrated ongoing hypertrophic scarring with associated pruritus (Figure 2 a,b). Due to logistical issues in getting the compression garments to his remote location, he had not yet received these. He was referred to the visiting dermatology out‐reach service for consideration of intralesional corticosteroid injections and ongoing follow‐up.

Hypertrophic or keloid scarring following TEN is not typical (Online Appendix 1) and while chronic pruritus has been reported to occur in 27%–73% of TEN patients,^1^ intense pruritus in the subacute re‐epithelialising phase followed shortly after by abnormal scarring has only rarely been described.^2^ Insults that result in delayed re‐epithelialisation (i.e. secondary infection, unrelieved skin pressure, prolonged immobility, surgical interventions, and delayed cessation of culprit drug) can increase the risk of hypertrophic and keloid scarring; as can genetic predisposition to abnormal scarring and self‐mutilation.^1^, ^2^, ^3^, ^4^ We speculate that several factors potentially contributed in our patient; including long half‐life of Paliperidone depot (median 25–49 days), concurrent dermatological diagnoses, secondary bacterial infection, multi‐nutrient deficiency and non‐Caucasian pigmented skin.^5^ It is possible that intense pruritus led to excoriation and secondary infection which may have further contributed to his hypertrophic scarring.

We suspect pruritus in the re‐epithelialising (sub‐acute) phase of TEN may be under‐reported. Nevertheless, in this patient, the temporal history of new intense pruritus over the anterior torso and proximal limbs, followed shortly after by the clinical and histopathological development of hypertrophic scarring in this distribution suggests pruritus may be an early sign of abnormal scarring. While the patient's face and distal limbs were equally affected by TEN, those sites did not develop pruritus or hypertrophic scarring. Accordingly, we feel our patient's pruritus was in keeping with the development of abnormal scarring, rather than simply wound healing. The clinical relevance of this case is twofold. Firstly, early management of pruritus is important to control excoriation and secondary infection which may increase the risk of abnormal scarring. Secondly, pruritus in the re‐epithelialising (sub‐acute) phase of TEN can help identify those patients who would benefit from early assessment with measurement for compression garments and consideration of other scar therapies. Larger, prospective, multi‐centric cohort studies are needed to accurately capture the sub‐acute sequelae of TEN.^1^

## CONFLICTS OF INTEREST

The authors have no conflicts of interest to declare.

## FUNDING INFORMATION

This letter received no specific grant from any funding agency in the public, commercial, or not‐for‐profit sectors.

## AUTHOR CONTRIBUTIONS

**Aaron J. Frederiks:** Conceptualisation (Equal); Writing – original draft (Lead); Writing – review & editing (Equal). **Jasmin D. Korbl:** Conceptualisation (Equal); Writing – original draft (Equal); Writing – review & editing (Equal). **Nima Mesbah Ardakani:** Conceptualisation (Equal); Writing – original draft (Equal); Writing – review & editing (Equal). **Fiona M. Wood:** Conceptualisation (Equal); Writing – original draft (Equal); Writing – review & editing (Equal). **Bernadette Ricciardo:** Conceptualisation (Equal); Supervision (Lead); Writing – original draft (Equal), Writing – review & editing (Lead).

## Supporting information

Supporting Information S1

## Data Availability

The data that support the findings of this study are available on request from the corresponding author. The data are not publicly available due to privacy or ethical restrictions.
