# Supplementary material for: Pruritus as an early sign of abnormal scarring in the re‐epithelialising phase of Toxic Epidermal Necrolysis
Source: Skin Health Dis. 2023 Feb 15;3(4):e217. doi: 10.1002/ski2.217 (PMC10395619; doi:10.1002/ski2.217)
Supplement: Supplementary file 1 — Supporting Information S1 [file SKI2-3-e217-s001.docx]

**Online Appendix 1**

The clinical course of TEN is typically 5-7 days of progressive epidermal detachment, followed by 1-3 weeks of re-epithelialisation ^1^. While hypertrophic or keloid scarring following TEN is not typical, a 2017 review article reported this in 10-40% of cases ^2^. Three case series were cited ^3-5^. The 2002 USA study found on burns clinic follow up that 1 of 11 (9%) paediatric patients had localised hypertrophic scarring to the upper back potentially from being in a supine position for a prolonged period ^5^. The 2005 Canadian study found 2 of 6 (33%) examined patients, both dark-skinned, had punctate keloids where staples had previously secured skin substitutes ^3^. The 2006 USA study found on patient questionnaire follow-up and telephone interview that 10 of 26 (38%) had scarring, though the type(s) of scarring was not specified ^4^. Conversely, a 2001 Italian study and 2003 Portuguese study both found 0 of 8 (0%) patients in their respective series developed hypertrophic scarring ^6,7^.

**References**

1. Estrella-Alonso A, Aramburu JA, González-Ruiz MY, Cachafeiro L, Sánchez Sánchez M, Lorente JA. Toxic epidermal necrolysis: a paradigm of critical illness. Revista Brasileira de terapia intensiva 2017;29:499-508.

2. Lee HY, Walsh SA, Creamer D. Long‐term complications of Stevens–Johnson syndrome/toxic epidermal necrolysis (SJS/TEN): the spectrum of chronic problems in patients who survive an episode of SJS/TEN necessitates multidisciplinary follow‐up. British journal of dermatology (1951) 2017;177:924-35.

3. Haber J, Hopman W, Gomez M, Cartotto R. Late outcomes in adult survivors of toxic epidermal necrolysis after treatment in a burn center. Journal of burn care & rehabilitation 2005;26:33-41.

4. Oplatek A, Brown K, Sen S, Halerz M, Supple K, Gamelli RL. Long-term follow-up of patients treated for toxic epidermal necrolysis. Journal of burn care & research 2006;27:26-33.

5. Sheridan RL, Schulz JT, Ryan CM, et al. Long-Term Consequences of Toxic Epidermal Necrolysis in Children. Pediatrics (Evanston) 2002;109:74-8.

6. Stella M, Cassano P, Bollero D, Clemente A, Giorio G. Toxic Epidermal Necrolysis Treated with Intravenous High-Dose Immunoglobulins: Our Experience. Dermatology (Basel) 2001;203:45-9.

7. Magina S, Lisboa C, Leal V, Palmares J, Mesquita-Guimarães J. Dermatological and Ophthalmological Sequels in Toxic Epidermal Necrolysis. Dermatology (Basel) 2003;207:33-6.
